# Peri-operative nurses’ knowledge and reported practice of pressure injury risk assessment and prevention: A before-after intervention study

**DOI:** 10.1186/1472-6955-11-25

**Published:** 2012-11-24

**Authors:** Sally Sutherland-Fraser, Elizabeth McInnes, Elizabeth Maher, Sandy Middleton

**Affiliations:** 1 Clinical Nurse Consultant for Peri-operative Practice Development, St Vincents Hospital, 390 Victoria Street, Darlinghurst, NSW, 2010, Australia; 2 EM Deputy Director, EMaher Research Assistant, Nursing Research Institute, St Vincents & Mater Health Sydney and Australian Catholic University, St Vincent's Hospital, Level 5, deLacy Building, 390 Victoria Street, Darlinghurst, NSW, 2010, Australia; 3 Director, Nursing Research Institute, St Vincents & Mater Health Sydney and Australian Catholic University. Director, Executive Office, Level 5, deLacy Building, 390 Victoria Street, Darlinghurst, NSW, 2010, Australia

**Keywords:** Pressure injury, Peri-operative, Nurses, Educational intervention, Prevention, Risk assessment

## Abstract

**Background:**

Patients are at risk of developing pressure injuries in the peri-operative setting. Studies evaluating the impact of educational interventions on peri-operative nurses’ knowledge and reported practice are scarce. The purpose of this study was to evaluate the effect of a multifaceted intervention on peri-operative nurses’ (a) knowledge of pressure injury risks, risk assessment and prevention strategies for patients in the operating suite; and (b) reported practice relating to risk assessment practices and implementation of prevention strategies for patients in the operating suite.

**Methods:**

A before-after research design was used. A convenience sample of all registered and enrolled nurses employed in two hospitals’ operating suites was recruited. A multifaceted intervention was delivered which comprised a short presentation, educational materials and reminder posters. A 48-item survey tool was completed pre-and post-intervention to measure self-reported knowledge and practice.

**Results:**

70 eligible peri-operative nurses completed both surveys. Post-intervention, statistically significant improvements were seen in knowledge of correct descriptions of pressure injury stages (p=0.001); appropriate reassessment for patients with a new pressure injury (p=0.05); appropriate actions for patients with an existing stage 1 (p=0.02) and stage 2 pressure injury (p=0.04). Statistical improvements were also seen in reported practice relating to an increase in the use of a risk assessment tool in conjunction with clinical judgement (p=0.0008); verbal handover of patients’ pressure injury risk status from the operating room nurse to the recovery room (p=0.023) and from the recovery room nurse to the postoperative ward nurse (p=0.045). The number of participants reporting use of non-recommended and recommended pressure-relieving strategies was unchanged.

**Conclusion:**

A multi-faceted educational intervention can improve some aspects of perioperative nurses’ knowledge and reported practice such as risk assessment practices but not others such as use of recommended pressure-relieving devices. Further research is required to ascertain effective interventions which improve all areas of practice and knowledge, particularly in the use of appropriate pressure-relieving devices in order to prevent pressure injuries in surgical patients.

## Background

Pressure injuries (also known as pressure ulcers and bedsores) are painful injuries of the skin or the underlying tissues, which form most often over a bony prominence as a result of unrelieved pressure, either in isolation or in combination with shearing forces [1]. Pressure injuries are rated in the top five adverse events in western countries [2], and the harm caused by them is considered to be largely preventable [2,3]. Pressure injuries can reduce quality of life [4-7] and have a negative social and psychological impact on the individual and their carers. Annual costs of medical treatment and costs of extended hospitalisations associated with having a pressure injury are high. These have been estimated at between £1-2 billion pounds in the United Kingdom [8]; $US11 billion in the USA [9] and in Australia $A285 million annually [10].

Reported pressure injury prevalence rates for acute care settings in Canada, Europe, Australia and North American range from 10–28% [2,11-14]. In relation to surgical patients reported rates vary, ranging from 12% to 66% depending on the study population, the type of surgery, the pressure-relieving support surfaces used and whether stage 1 (least severe) pressure injuries are included [15-18]. This indicates that surgical patients are vulnerable to elevated pressure injury risk. Over the last decade, a number of studies have examined nurses’ knowledge and practice of pressure injury risk assessment (use of assessment tools and clinical judgement), prevention and management. These studies have been conducted in a variety of clinical settings and have employed different methods including knowledge and attitude tests, surveys, interviews and medical record audits [19-24]. While some studies report good levels of knowledge amongst nurses about pressure injury risks and appropriate pressure injury risk assessment and prevention practices [21,22], some studies report that nurses’ knowledge is inadequate, does not reflect the current evidence and that evidence-based strategies are not always applied in clinical practice [19,23-25]. An Australian survey of 2113 registered nurses found that only 30% documented assessment and treatment of pressure injuries; 53% followed repositioning guidelines and over 50% used the outdated practice of water-filled gloves to prevent pressure by over a half [26]. A Belgian study of 22 hospitals found only 17.5% of at-risk patients were appropriately allocated a dynamic mattress system [27]; a Swedish study conducted in a large teaching hospital found that the majority of those at risk for pressure injuries did not get evidence-based care [28]; while only half of eligible patients across 89 Dutch institutions were allocated a pressure-relieving support surface and less than one-third were regularly repositioned [29]. These studies and others have also found inadequacies and gaps in nurses’ documentation of pressure injury risk assessment, and prevention measures used [19,30-33].

To address these evidence-practice gaps, a number of studies have evaluated the impact of different strategies on nurses’ knowledge and practice, although no studies were found that had specifically targeted peri-operative nurses. For example, following a 40-minute education session (didactic presentation of slides with question and answer) the knowledge of nurses working in an acute hospital setting in the United States improved when tested immediately after the education [34]. However, no follow-up testing was done so knowledge retention was not ascertained. A follow-up study examined the effects of a three and a half hour multifaceted education program (combining didactic and interactive delivery of educational material, video and role play) on nurses’ knowledge of pressure injury prevention and achieved a significant improvement in nurses’ knowledge scores at three months post-intervention [35]. A large Canadian study found nurses’ knowledge improved following the implementation of pressure injury clinical practice guidelines using a behaviour change model which consisted of comprehensive education program (combining videos, tutorials and self-directed material), a full day of education for clinical mentors, and a computer-based system to guide the nurses’ practice with risk assessment and selection of pressure injury prevention strategies [36]. Prentice and Stacey [25] found that introduction of pressure injury clinical practice guidelines combined with a comprehensive, multifaceted education pressure injury program, for junior medical and nursing staff that included peri-operative nurses, significantly reduced pressure injury prevalence. Other combinations of multi-faceted strategies have similarly reported improvements in knowledge and practice [31,37]. Some studies have reported mixed results of strategies targeting nurses’ knowledge and documentation that included an educational component, with improvements on some domains such as documentation practices [38] or on reported use of risk assessment and prevention strategies [39] or no statistically significant improvements at all [21].

Considering that a number of studies and systematic reviews have found that multi-faceted interventions are effective in changing practice for a number of areas of clinical care [40,41], this type of intervention also holds promise for improving knowledge of pressure injury evidence-based care amongst perioperative nurses, an area that has been largely unevaluated. We report here the effects of a multifaceted intervention aimed at improving (a) perioperative nurses’ knowledge and reported practice with pressure injury risk assessment and initiation of pressure prevention strategies for peri-operative patients. Addressing deficiencies in knowledge and practice of pressure injury prevention in the peri-operative setting is particularly important because of the vulnerability of surgical patients to an elevated risk of postoperative pressure injuries [29,42-44]. We hypothesized that a multifaceted intervention consisting of group education; reminder posters and dissemination of educational resources would improve peri-operative nurses’ knowledge and reported practices relating to pressure injury assessment and prevention.

## Methods

### Aims

The aims of the study were twofold: to determine the effect of an educational intervention on

(a) peri-operative nurses’ knowledge of pressure injury risk assessment and prevention strategies for patients in the operating suite; and (b) peri-operative nurses’ reported practice regarding pressure injury risk assessment and prevention strategies for patients in the operating suite.

### Design

This research was designed as a pre-post intervention study.

### Setting

The study was conducted in the operating suites of two large metropolitan hospitals in Sydney, between April 2009 and February 2010. Hospital 1 is a 305-bed tertiary-referral facility in the public sector and Hospital 2 is a 195-bed private facility.

### Participants and procedure

A convenience sample of all eligible registered and enrolled nurses employed in the participating operating suites was recruited for the survey. Nurses working in direct-patient care roles in the operating theatre setting and employed on a full-time or part-time basis were eligible for inclusion (part-time was defined as minimum of two shifts per fortnight and the expected duration of the study was nine months). Nurses employed for short clinical placements to the operating suites; on a casual basis or employed for specific surgeons’ lists were not eligible as they would not be present for the entire study period. An invitation letter and a pre-intervention questionnaire was attached to all eligible nurses’ payslips six weeks before the educational intervention. Completion of the questionnaire was considered implied consent to participate in the study.

### Intervention

The multifaceted intervention, Pressure Ulcer Prevention Program (PuPP), was developed by the first author, who is a registered nurse with 30 years experience in the peri-operative setting. Core content was derived from the latest national pressure injury prevention guidelines at the time of the study [45] and from research literature on pressure injury risks for peri-operative patients [9,17,18,44,45]. The intervention (Table 1) was informed by the findings from a systematic review that multifaceted educational interventions are more effective in terms of practice change than single interventions alone [46].

**Table 1:** PuPP intervention elements

**Intervention**	**Contents**
30-minute audio-visual presentation outlining pressure injury:	a. Aetiology
	b. Staging
	c. Risk factors for peri-operative patients
	d. Risk assessment & documentation requirements
	e. Peri-operative pressure prevention strategies
	f. Evidence based resources, policies & guidelines
Resource folder and companion CD:	a. Slides from presentation
	b. Evidence-based references
	c. Current policies and guidelines
Reminders	a. Verbal reminders of audio-visual presentation key points provided at staff meetings
	b. Visual reminders of audio-visual presentation key points provided by four series of colour posters on prominent display throughout the operating suite over 8 weeks between survey periods (nb: a new series of four posters displayed every 2 weeks) addressing:
	- The importance of a preoperative risk assessment score;
	- Recommended pressure prevention devices;
	- Alerts regarding non-recommended pressure prevention devices;
	- Intraoperative documentation requirements

PuPP comprised three elements: i) a 30 minute presentation by the first author on pressure injury risks and prevention strategies for the peri-operative setting; ii) a companion folder of educational materials, additional reference material and policy documents; and iii) a series of reminder posters on the key points from the presentation. The latter two resources were placed in the peri-operative workplace and all nurses would have been exposed to these resources in the course of the working day. The intervention was implemented from August to December 2009, approximately six weeks after distribution of the pre-intervention survey.

### Data collection

The pre-intervention survey was conducted April–July 2009 and the post-intervention survey was conducted October 2009–February 2010. Secure survey collection boxes were provided for anonymous return of completed questionnaires and consent forms in both operating suites. The same process was repeated for the post-intervention survey six weeks after the educational sessions were held. There were three prompts at each time period to remind non-responders to complete their questionnaires.

The self-administered standardised questionnaire was developed for the study by the first author, with reference to content in similar questionnaires that have assessed nurses’ pressure injury knowledge and practice [21,47,48] and also with reference to systematic reviews and guidelines [1,49]. The content of the questionnaire was designed to reflect the content of the intervention. A group of nurse educators practising in peri-operative settings reviewed the tool for face validity and piloted the tool. The purpose of the survey was to assess any changes in reported knowledge and practice before and after the multi-faceted intervention.

The questionnaire comprised 48 items (closed and open-ended questions). Demographic data was collected on employment details, practice areas and peri-operative roles and year of first nursing qualification. Reported knowledge was measured at both time points by questions on: a) pressure injury staging; b) definitions of pressure injury stages according to the NPUAP (2007) stages and local policy; and c) post-operative nursing actions to manage a patient with a new stage 1 heel pressure injury and a new stage 2 pressure injury on the buttocks.

Reported practice was measured at both time points by questions on: a) methods (for example, risk assessment tools and clinical judgement) used for pressure injury risk assessment; b) selection of positioning and pressure relieving devices most frequently used for pressure prevention in the peri-operative setting from a list which included recommended devices, non-recommended devices and devices for which there is no evidence of effectiveness, and c) the use of pressure injury risk assessment scores during clinical handover.

Five case scenarios were used as a basis for questions related to reported practice (Table 2). In Case 1, we asked nurses to indicate their likelihood of completing the PI risk assessment when the patient’s condition has changed, and in Case 2, when pressure damage is identified. We also asked nurses to indicate their likelihood of viewing the PI risk score in the patients notes; and the likelihood of reporting the PI risk score at three points of clinical handover during the peri-operative episode; namely on admission of a patient to the operating suite (Case 3); on transfer of a patient to the recovery room (Case 4); and on discharge of a patient from the recovery room to the postoperative ward (Case 5) (Table 2). Some items on the questionnaire were dichotomous (yes/no); while others used a five-point Likert scale ranging from highly likely (1) to highly unlikely (5).

**Table 2:** Peri-operative nursing practice case study scenarios

**Case Study**	**Scenario**
Case Study 1	Patient condition changes during the peri-operative period –indicate likelihood of completing the pressure injury (PI) risk assessment
Case Study 2	Pressure injury is identified during the peri-operative period – indicate likelihood of completing the PI risk assessment
Case Study 3	Patient admitted to operating suite – indicate likelihood of:
	a) viewing the PI risk assessment score in the notes; and
	b) reporting the PI risk assessment score during this clinical handover
Case Study 4	Patient transferred from operating room to recovery room – indicate likelihood of:
	a) viewing the PI risk assessment score in the notes; and
	b) reporting the PI risk assessment score during this clinical handover
Case Study 5	Patient discharged from recovery room to postoperative ward – indicate likelihood of:
	a) viewing the PI risk assessment score in the notes; and
	b) reporting the PI risk assessment score during this clinical handover

### Ethical considerations

The protocol for this research project was approved by the Human Research Ethics Committee of the Australian Catholic University and hospital sites. Consent was implied by return of questionnaire.

### Data analysis

Analysis was undertaken on those who had completed both the pre and post-intervention questionnaires. Sample characteristics and paired data were analysed using SPSS (Version 17.0). Frequencies were obtained for all variables. Significance tests were conducted using McNemar’s test for analyses of paired categorical data and the paired *t*-test to analyse any changes in mean scores relating to knowledge and practice post-intervention that was measured using Likert scales.

## Results

Questionnaires were distributed to all eligible nurses at both operating suites (pre-intervention n=146; post-intervention n=143) and we received a total of 90 surveys back from the pre-intervention mail out (response rate 62%) and 90 surveys from the post-intervention mail out (response rate 63%).

### Sample

Seventy nurses participated in both surveys, and these comprised the sample for analysis. The majority of these nurses were registered nurses (n=61, 88%). The number of years since first nursing qualification ranged from 2 to 44 years with the mean number of years being 16 (sd 11). The majority of nurses qualified from 1986 when nursing education in Australia was transferred from the hospital to the university sector (n=45, 78%). More than two-thirds of nurses were employed full-time. Table 3 shows that the group almost equally comprised of nurses practising in the intra-operative roles (circulating nurse and instrument nurse) and those in either anaesthetic or post-anaesthetic recovery roles. Only 20% had received education on pressure injury in the past 2 years and most of the sample either could not remember when they had last read anything on pressure injury risk (59%) and management, or reported that they had never read anything on pressure injury risk and management (10%).

**Table 3:** Participant demographics and reported previous pressure injury education (n=70)

	**n**	**%**
**Designation ^**		
Registered nurse	61	88
Enrolled nurse	8	12
**Employment**		
Full time	49	70
Part time	21	30
**Usual shifts**^**#**^		
Rotating roster	39	56
AM Weekdays	28	40
PM Weekdays	14	20
Nights or weekends only	4	6
**Main area of practice**^**#**^		
Anaesthetics	22	31
Intraoperative roles	38	54
Post-anaesthetic recovery	16	23
Education	6	9
**First Nursing Qualification ^**		
Before 1986^~^	13	22
From 1986	45	78
**Received education on PI within last 2 years**	14	20
**When last read anything on the risks and management of PI**		
< 3 months	2	3
4 - 12 months	3	4
> 1 year	17	24
Can’t recall	41	59
Never	7	10

^ Data missing.

^#^ Where totals add to > 100%, more than one answer was possible.

^~^ 1986 was the date Australian nursing education moved to the University sector.

### Peri-operative nurses’ reported knowledge and actions

A significantly higher proportion of participants post-intervention accurately described the four PI stages according to the NPUAP staging system (increasing from 52% to 83% post-intervention, *X*^2^(3)=30.12, p=0.001). For a patient with a new stage 1 PI on the heel, a significantly higher proportion of participants post-intervention reported that they would re-assess the patient’s PI Risk Assessment score, increasing from 41% to 57% (*X*^2^(1)=4.71, p=0.05) (Table 4). Of the non-recommended actions, post-intervention significantly fewer nurses reported they would rub or massage the affected area for a patient with a new stage 1 heel pressure injury (decreasing from 31% to 14%, *X*^2^(1)=0.40, p=0.02) or a new stage 2 pressure injuries on the buttocks (12% to 3%, *X*^2^ (1)=8.08, p=0.04). All other results were non-significant (Table 4).

**Table 4:** Peri-operative nurses’ reported knowledge and actions pre and post-intervention (n=70)

	**Pre-intervention**		**Post-intervention**		**P-value**^**+**^
**Pressure injury stages^**	**n**	**%**	**n**	**%**	
Correctly describe PI stages	36	52	57	83	**0.001**
Correctly match PI stages to descriptions	57	85	61	91	0.581
**Actions for patient with new stage 1**^**β**^**PI on heels**^**#**^	**n**	**%**	**n**	**%**	
Reassess PI risk assessment (PI RA) score	29	41	40	57	**0.05**
Notify the nurse at handover	63	90	67	96	0.289
Complete an incident report	32	46	29	41	0.711
Elevate affected area onto offloading device	34	49	39	56	0.458
Rub / massage the area^€^	22	31	10	14	**0.02**
Reposition affected area onto donut air-pillow^€^	38	54	35	51	0.85
**Actions for patient with new stage 2**^**β**^**PI on buttocks**^**#**^**^**	**n**	**%**	**n**	**%**	
Reassess PI RA score	33	48	42	60	0.16
Notify the nurse at handover	64	93	64	93	1.00
Complete an incident report	41	59	49	71	0.152
Reposition patient onto their side	45	65	52	75	0.210
Rub / massage the area^€^	8	12	2	3	**0.04**
Reposition affected area onto donut air-pillow^€^	17	25	22	31	0.67

^+^ Chi-square test of association was used for proportions.

^ Data missing.

^***β***^ NPUAP Pressure Ulcer Staging System.

^#^ Where totals add to > 100%, more than one answer was possible.

^€^ Contraindicated nursing actions.

### Peri-operative nurses’ reported practice

Table 5 shows that a significantly higher proportion of nurses post-intervention would use a PI Risk Assessment tool to assess patients for PI risk (increasing from 12% to 40%, *X*^2^(1)=3.26, p=0.0001), and would use both a PI risk assessment tool and clinical judgement (increasing from 9% to 27%, *X*^2^(1)=0.01, p=0.008). Pillows, gel pads and gel overlays were the three most frequently reported devices used for pressure prevention in the peri-operative setting both pre-and post-intervention (ranging from 61% to 96%). The numbers of those using non-recommended devices or devices with no evidence to support their use did not change. There was a statistically significant mean increase in the likelihood that the patient’s PI risk assessment score would be verbally reported during clinical handover from the operating room nurse to the recovery room (t, _1,49_=2.36, p=0.023) and significantly higher mean increase in participants reporting that it was likely that the PI score would be verbally reported from the recovery room nurse to the postoperative ward nurse (t, _1,42_=2.09, p=0.045). There were no other significant results (Table 6).

**Table 5:** Peri-operative nurses’ reported practice pre- and post-intervention (n=70)

	**Pre-intervention (%)**		**Post-intervention (%)**		**P-value**^**+**^
**Pressure injury risk assessment method**^#^^	**n**	**%**	**n**	**%**	
Risk assessment tool	7	12	24	40	**0.000**
Clinical judgement alone	55	92	55	92	1.000
Risk assessment tool & clinical judgement	6	9	19	27	**0.008**
**Devices used for pressure injury prevention in operating room**^**#**^**^**	**n**	**%**	**n**	**%**	
Recommended devices					
Pillows	64	96	60	90	0.289
Gel (rings/pads/cushions)	51	76	52	78	1.000
Gel (table overlay)	41	61	46	69	0.383
Foam (rings/pads/cushions)	27	40	28	42	1.000
Replacement mattress (fluidised gel)	14	21	18	27	0.388
Non-recommended devices					
Donut air-pillow	20	30	17	25	0.581
Water-filled gloves	2	3	2	3	1.000
No current evidence					
Towels	19	28	16	24	0.607
Blankets	12	18	10	15	0.791
Beanbag	11	16	11	16	1.000

^+^ Chi-square test of association was used for proportions.

^#^ Where totals add to > 100%, more than one answer was possible.

^ Data missing.

**Table 6:** Peri-operative nurses’ reported practice relating to case studies (n=70)

	**Pre-intervention Mean (SD)** (**Range)**	**Post-intervention Mean (SD) (Range)**	**P-value**^**+**^
**Pressure injury risk assessment**			
Case study 1: Risk assessment score completed when patient’s condition changes	2.62 (1.56) (1–5)	2.0 (1.08) (1–5)	0.136
Case study 2: Risk assessment score completed when pressure damage is identified	2.07 (1.49) (1–5)	1.29 (0.47) (1–5)	0.085
**Clinical handover from ward nurse (admission to operating room)**			
Case study 3: Risk assessment score is:			
Viewed in patient notes	3.37 (1.55) (1–5)	2.80 (1.47) (1–5)	0.069
Verbally reported	3.33 (1.46) (1–5)	2.96 (1.40) (1–5)	0.120
**Clinical handover from operating room nurse (transfer to recovery unit)**			
Case study 4: Risk assessment score is:			
Viewed in patient notes	3.39 (1.52) (1–5)	3.09 (1.43) (1–5)	0.223
Verbally reported	3.40 (1.55) (1–5)	2.86 (1.46) (1–5)	**0.023**
**Clinical handover from recovery room nurse (discharge to ward)**			
Case study 5**:** Risk assessment score is			
Viewed in patient notes	3.09 (1.49) (1–5)	2.64 (1.43) (1–5)	0.142
Verbally reported	3.03 (1.49) (1–5)	2.52 (1.35) (1–5)	**0.045**

^+^Paired sample t-tests were used.

## Discussion

This study is the first to examine the effects of a multifaceted intervention on peri-operative nurses’ knowledge and practice with regard to pressure injury risk assessment and pressure injury prevention strategies. There were mixed results in regard to the impact of our intervention on aspects of self-reported knowledge and practice, with improvements not occurring in the desired direction. These results have implications for patient care and safety in the peri-operative setting. Similar findings have also been reported in similar studies that have targeted nurses in other settings [21,49].

There were significant improvements post-intervention in participants’ ability to correctly describe pressure injury stages. There were also improvements in relation to knowledge of the correct management of patients, with significantly more nurses reporting they would re-assess risk assessment score for a patient with a new pressure injury stage 1 on the heel (recommended practice). In addition, significantly fewer nurses reported post-intervention they would use the non-recommended action of rubbing or massaging the affected area for a patient with a new pressure injury. There were, however, no improvements in knowledge regarding notification of the nurse at handover of new pressure injuries (stage 1 or 2); completion of an incident report and repositioning the patient onto their side (recommended practice). It is of concern from a patient safety perspective that a multifaceted and comprehensive evidence-based intervention, specifically targeted at nurses who are involved at all stages of the patient peri-operative journey, did not increase their knowledge on these factors.

Encouragingly, two measures of nurses’ reported practice showed improvement following the intervention. Significantly more nurses post-intervention reported using a tool in combination with clinical judgement in line with best practice [1]. This is noteworthy because while clinical judgement is recognised as an important component of risk assessment, research has shown that clinical judgement in isolation is not an adequate or reliable predictor of pressure injury risk [50,51]. Judgement of a patient’s risk status is an important basis for deciding to implement preventive measures [37]. Reasons for this favourable change occurring, might be because risk assessment documentation is included within a patient’s medical notes. Thus, once the intervention raised awareness about the importance of risk assessment, the risk assessment form provided an ongoing and salient reminder of need to complete an assessment.

There were no changes in relation to knowledge or practice of use of non-recommended pressure prevention devices. Ongoing use of non-recommended actions for pressure injuries such as the use of donut air-pillows, which have been long discredited, has also been reported by other researchers [23,26,52]. The persistence of these practices are thought to have their source in education from past decades, when these devices were more commonly advocated as part of nursing care and pressure prevention [53]. Considering that most of our sample reported obtaining their first nursing qualifications after 1986, meaning they had undergone university undergraduate nursing education it could be expected that they would have more current knowledge of the evidence. Yet, 59% of our sample reported that they could not recall when they had last read anything on pressure injury risks and management prior to our intervention. This indicates that recency of education through the tertiary system is insufficient for overcoming non-recommended practices and that pressure injury education needs to be ongoing within facilities. Another issue that may have impacted on our results, is one that is now commonly reported in respect of patient safety issues. That is that there are now many competing campaigns to improve patient safety and quality of care within facilities and this may lead to messages being diluted or health care professionals’ experiencing message fatigue [54]. The other possibility is that the continued availability of non-recommended devices within practice settings contribute to their continued use, years after such devices have been discredited and are no longer recommended [49,55]. Furthermore, implementation science research suggests that not only is it difficult to implement new practices, it can be equally challenging to undo practices that have been in place for some time [56].

In addition, there was no reported increase in terms of perioperative nurses use of recommended pressure-relieving devices. However, for some recommended devices use was high at both time points (90% for one recommended device and nearly 80% for another). It is likely too that surgeons’ and anaesthetists’ preferences for particular pressure-relieving devices may be an influential factor in use of particular devices. This suggests that future interventions focused on disseminating evidence-based recommendations should target the entire peri-operative team.

Our results indicated that there are reported practices and areas of knowledge that are harder to shift in the desired direction in line with the evidence, a situation that has been reported in nursing implementation research [56]. Studies have found nurses’ improved knowledge does not necessarily lead to improved practice or changed behaviours [23,57], and other educational interventions targeting pressure injury prevention have found similar results among other groups of nurses [39,58]. Educational strategies that are combined with either audit and feedback [31,59]; the introduction of recommended pressure relieving devices [18]; and simultaneous introduction of guidelines [25] may increase the effectiveness of educational interventions. A top-down push by nursing leadership to support changes in nurses’ practice can also be effective [36].

Deficiencies in knowledge and practice of aspects of pressure injury prevention in the peri-operative setting despite a multi-faceted intervention is concerning because surgical patients are at high risk of postoperative pressure injuries. Further research is therefore warranted to investigate the factors that influence peri-operative nurses and other peri-operative team members decision-making in respect of evidence-based care, in order to better inform the development of future targeted interventions.

### Strengths and limitations

The strengths of this study include the survey size, which was multi-site, with peri-operative nurses participating from two large metropolitan hospitals, and the good response rate of 62% pre-intervention and 63% post-intervention which is comparable with other surveys of hospital clinicians [60]. We calculated a post-hoc power calculation based on the results for change of difference in reported practice relating to undertaking risk assessment. This calculation found that a sample size of 70 has 95% power to detect a difference between means of 0.5 at the 0.05 level (2-tailed). We concluded that this was reasonable power to detect this difference in mean score. However, this was a convenience sample and the main objective of the study was to evaluate the impact of an intervention on reported rather than actual knowledge and practice. The intervention was multifaceted, in line with current evidence [46], and was developed by peri-operative nurse educators and based on evidence-based guidelines. The post-intervention survey was performed between one and three months after the intervention, so we cannot report on the nurses’ long-term knowledge retention or the sustainability of the nurses’ reported practice change beyond that period. We sampled and targeted only peri-operative nurses because generally they are present at all stages of the patient journey in the peri-operative setting. However, our results, particularly those relating to use of recommended and non-recommended pressure-relieving devices, suggests that the whole peri-operative team should be exposed to educational interventions. This should include surgeons and anaesthetists.

## Conclusion

Our study found that educational interventions can improve some aspects of nurses’ knowledge and reported practice in peri-operative settings. Practices relating to the use of non-recommended pressure-relieving support surfaces may be more difficult to change and require additional intervention strategies such as audit and feedback and simultaneous introduction of guidelines and recommended pressure-relieving devices. Education on pressure injury risks, risk assessment and prevention strategies should be included in orientation programs and with ongoing education in the peri-operative setting. Where practice is influenced by team-based decisions, such as in the operating room, interventions should consider targeting all members of the peri-operative team. Further research is needed to investigate whether improvements in peri-operative nurses’ knowledge and reported practice are associated with improvements in nurses’ actual practice and whether this in turn affects pressure injury incidence in the post-operative period.

## Abbreviation

PI: pressure injury.

## Competing interests

The authors declare that they have no competing interests.

## Authors’ contribution

SS-F conceived the study topic. SS-F and SM finalised the study design. EM and SM advised on protocol details including recruitment. SS-F, EM and EMaher prepared the framework of this manuscript. SS-F and EMaher collected and inputted data. SS-F, EMaher and EM analysed the data. SS-F and EM led revisions of the paper. All authors read and approved the final manuscript.

## Pre-publication history

The pre-publication history for this paper can be accessed here:

http://www.biomedcentral.com/1472-6955/11/25/prepub
